# Two decades of experience of the Fabry Outcome Survey provides further confirmation of the long-term effectiveness of agalsidase alfa enzyme replacement therapy

**DOI:** 10.1016/j.ymgmr.2025.101215

**Published:** 2025-04-11

**Authors:** Uma Ramaswami, Guillem Pintos-Morell, Christoph Kampmann, Kathleen Nicholls, Dau-Ming Niu, Ricardo Reisin, Michael L. West, Christina Anagnostopoulou, Jaco Botha, Dalia Jazukeviciene, Jörn Schenk, Derralynn A. Hughes, Roberto Giugliani

**Affiliations:** a Royal Free London NHS Foundation Trust, University College London, Pond Street, London NW3 2QG, UK; b Vall d'Hebron Institute of Research (VHIR), Vall d'Hebron Barcelona Hospital Campus, Passeig Vall d'Hebron 119-129,08035 Barcelona, Spain; c Johannes Gutenberg School of Medicine, University of Mainz, Saarstraße 21, 55122 Mainz, Germany; d The Royal Melbourne Hospital and the University of Melbourne, 300 Grattan Street, Parkville, VIC 3052, Australia; e Taipei Veterans General Hospital, No. 201, Section 2, Shih-Pai Road, Taipei 112, Taiwan; f Hospital Británico de Buenos Aires, Perdriel 74, C1280AEB Cdad., Buenos Aires, Argentina; g Department of Medicine, Dalhousie University, 5849 University Avenue, Halifax, NS B3H 4R2, Canada; h Takeda Pharmaceuticals International AG, Thurgauerstrasse 130, 8152 Glattpark (Opfikon), Zurich, Switzerland; i Department of Genetics, UFRGS, Medical Genetics Service, HCPA, INAGEMP, Dasa Genomica and Casa dos Raros, Rua Sao Manoel 730, Porto Alegre, RS 90610-261, Brazil

**Keywords:** Registry data, Clinical outcomes, Agalsidase alfa, Fabry disease, Long-term data

## Abstract

**Background:**

Analyses of up to 20 years of data from the Fabry Outcome Survey (FOS) assessed the long-term effectiveness of agalsidase alfa enzyme replacement therapy.

**Methods:**

The impact of agalsidase alfa treatment on renal, cardiac, morbidity, and mortality outcomes in FOS was compared with untreated external Fabry disease (FD) cohorts.

**Results:**

A total of 2171 FOS patients (1014 men, 919 women, 163 boys, 75 girls) received agalsidase alfa (median [range] duration of treatment: 5.38 [0.0–20.8] years). Annual rates of decline in estimated glomerular filtration rate improved in treated patients versus untreated external cohorts regardless of sex or baseline urinary protein levels. Annual left ventricular mass index rates were stable in treated patients regardless of sex or baseline left ventricular hypertrophy status, and better than in untreated external cohorts. The mean age at which 50 % of patients had their first composite morbidity event was later in the agalsidase-alfa-treated population than in the untreated external cohort (51.7 vs 41 years [males]; 60.8 vs 53 years [females]). After 24 months of treatment, the probability of a composite morbidity event was ∼34 % in treated patients and ∼ 45 % in untreated patients. Treated patients were older at death than untreated patients (mean [range]: 61.7 [26.2–87.6] vs 50.3 [34.5–70.1] years). The mean age at which 50 % of male patients were still alive was higher in treated patients than in untreated external cohorts (75.5 vs 60.0 years).

**Conclusions:**

Long-term treatment with agalsidase alfa may provide renal, cardiac, and overall survival protection in FD.

## Highlights

• The agalsidase alfa FOS cohort (*n* = 2171) was compared with untreated external cohorts
• Agalsidase alfa improved renal outcomes regardless of sex or baseline proteinuria
• Agalsidase alfa stabilized cardiomyopathy regardless of sex or LVH at baseline
• Agalsidase alfa delayed composite morbidity events and mortality
• Agalsidase alfa (5.38 [0–20.8] years) provided renal, cardiac and survival protection

## 1 Introduction

Fabry disease (FD) is a rare X-linked lysosomal storage disease caused by defective variants in the *GLA* gene that encodes the alpha-galactosidase A (α-Gal A) enzyme [1,2]. Reduced or absent α-Gal A enzyme activity leads to an accumulation of glycosphingolipids, particularly globotriaosylceramide (Gb_3_) and globotriaosylsphingosine (lyso-Gb_3_), throughout the body resulting in cell, tissue, and organ damage [1,3,4]. For some individuals with missense variants of *GLA*, misfolding and abnormal intracellular retention of α-Gal A enzyme causes a proteostasis reaction mechanism called the unfolded protein response, which reduces the production or increases the catabolism of unfolded proteins in the endoplasmic reticulum. The pathogenic intracellular trafficking of such missense α-Gal A variants results in a milder phenotype (AGALopathy) with less pronounced lysosomal storage than in classic FD [5].

Patients with FD typically experience disease-related complications including neuropathy, renal and cardiac dysfunction/failure, cerebrovascular events, and reduced life expectancy [1,6,7]. Prevalence estimates of FD range from 1 in 117,000 live births to 1 in 11,854 live births [[8], [9], [10]]; however, these estimates are considerably higher when non-classic variants are included [11].

Enzyme replacement therapy (ERT) with recombinant α-Gal A has been shown to reduce Gb_3_ and lyso-Gb_3_ concentration in blood, urine, and tissue and to have a positive influence on pain-related quality of life outcomes, cardiac morphology, and renal function [12,13]. Five ERT formulations for FD are currently available: agalsidase alfa (Replagal®, Takeda Pharmaceuticals International AG) [14], agalsidase beta (Fabrazyme®, Sanofi Genzyme) [15], two agalsidase beta biosimilars (agalsidase beta BS, JCR Pharmaceuticals Co., Ltd., and Sumitomo Pharma Co., Ltd.; Fabagal®, ISU ABXIS), and pegunigalsidase alfa (Elfabrio®, Chiesi Farmaceutici S.p.A.). Additionally, treatment with the pharmacologic chaperone therapy migalastat (Galafold®, Amicus Therapeutics) is approved for patients with amenable *GLA* variants [16,17].

Real-world evidence generated from disease registries is valuable to physicians and patients as well as to regulatory and payer authorities, especially for rare diseases such as FD, where clinical trials may have included a relatively small number of participants and may have stringent inclusion and exclusion criteria. The Fabry Outcome Survey (FOS) is an international, multicenter, observational, physician-directed registry initiated in Europe in 2001 by Transkaryotic Therapies Inc. (TKT) Europe-5S AB and subsequently expanded worldwide and sponsored by TKT, then by Shire Human Genetic Therapies, and later by Takeda Pharmaceuticals International AG [7,11,18]. FOS stopped recruiting patients in 2022. FOS aims to provide long-term data on the effectiveness and safety of approved FD-specific therapies (mainly agalsidase alfa) to broaden the understanding of this rare disease and to contribute to better clinical management [11].

Agalsidase alfa treatment has previously been reported to be associated with slowing progression of renal and cardiac manifestations, with reduction of neuropathic pain and gastrointestinal symptoms, and with reduced mortality, in FD [[19], [20], [21], [22], [23], [24], [25], [26], [27], [28], [29], [30], [31]]. These studies generally do not include a comparable, untreated cohort; as also seen in FOS, untreated patients tend to be less severely affected by FD and have fewer follow-up assessments than treated patients [32]. However, long-term data on FD-related outcomes in untreated/natural history populations are available from other published studies [[33], [34], [35], [36]] and have been used previously as comparator groups to estimate treatment benefit [20].

We report an analysis of the impact of agalsidase alfa treatment on renal, cardiac, morbidity, and mortality outcomes in patients with FD by using up to 20 years of data from the FOS registry, compared with untreated patients from previously published studies. These analyses build on a previous report by Beck and colleagues on outcomes with 5 years of agalsidase alfa treatment [37].

## 2 Methods

### 2.1 FOS registry and patients

FOS (ClinicalTrials.gov identifier: NCT03289065) is a worldwide disease registry with up to 20 years of data from treated and untreated patients who have a confirmed diagnosis of FD.

Participation in FOS was approved by the ethics institutional review boards of each participating center, and all patients provided written informed consent. While FOS was open, it was compliant with relevant local regulations and best practices.

Male and female patients with a diagnosis of FD and who were either receiving agalsidase alfa at the approved dose or had not received any ERT before FOS entry were eligible for inclusion. Additionally, from 2016, patients receiving agalsidase beta or migalastat were eligible for enrollment, although these patients were excluded from the current set of analyses; patients who were treated with agalsidase alfa and switched to agalsidase beta or migalastat were excluded from these analyses as well, from the time of switching. For the purpose of this analysis, treated FOS patients were defined as patients who had received agalsidase alfa for any length of time (and not necessarily while enrolled in FOS) and who had not received agalsidase beta or migalastat before or after initiating agalsidase alfa.

FOS data used for these analyses were from database inception in 2001 through to August 3, 2021, using methods previously described [18,21,32,38]. Baseline values were defined as the values obtained at the closest date to starting agalsidase alfa (within 12 months, with a preference towards values obtained before initiating treatment) in treated patients. Follow-up time was defined as the time from the first to the last recorded treatment with agalsidase alfa.

**Fig. 1:**
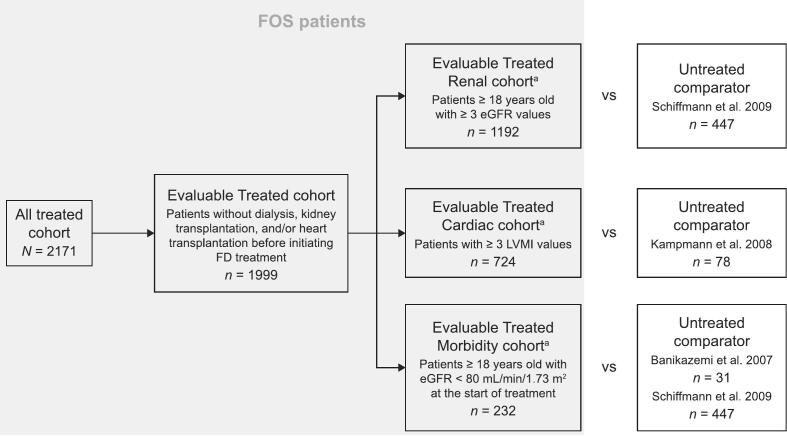
FOS patient cohorts and untreated external cohorts used throughout the current set of analyses. eGFR, estimated glomerular filtration rate; FOS, Fabry Outcome Survey; LVMI, left ventricular mass index. ^a^ FOS patients were included in every sub-cohort for which they filled the inclusion criteria.

For data analysis purposes, FOS patient cohorts were defined as the All Treated cohort, including all FOS patients who started agalsidase alfa before or at FOS registry entry, and the Evaluable Treated cohort, a subset of the All Treated cohort that excluded patients who had undergone dialysis, kidney transplantation, and/or heart transplantation before initiating FD treatment (Fig. 1).

The Evaluable Treated cohort was further subdivided – depending on the nature of the data analyzed – into three overlapping groups: the Evaluable Treated Morbidity cohort, including patients ≥ 18 years old with baseline estimated glomerular filtration rate (eGFR; calculated using the Modification of Diet in Renal Disease [MDRD] equation) < 80 mL/min/1.73 m^2^ at the start of treatment, the Evaluable Treated Renal cohort, including patients ≥ 18 years old at the start of treatment who had ≥ 3 eGFR measures (at least baseline and two post-baseline time-points), and the Evaluable Treated Cardiac cohort, including patients who had ≥ 3 LVMI measures (at least baseline and two post-baseline time-points).

For comparison with treated patients, published untreated patient data were derived from the FD retrospective chart study by Schiffmann et al. [35] (for mortality, morbidity, and the progression of renal impairment), the prospective study by Kampmann et al. [34] (for cardiac changes over time), and the randomized study by Banikazemi et al. [33] (for time from baseline to onset of first clinical event, e.g. death, cardiac, renal).

For genotype/phenotype-related outcomes, the FOS population was subdivided into patients with classic FD and those with non-classic FD. FD variants were assigned to a phenotype based on published sources [28,[39], [40], [41], [42], [43], [44], [45], [46], [47], [48], [49], [50], [51], [52], [53]], the fabry-database.org GLA variant database [54], and the International Fabry Disease Genotype-Phenotype Database (dbFGP) [55]. Non-classic FD variants included N215S and IVS4+919G>A [56] but excluded D313Y [57]. Variants of unknown significance, those likely to be benign or nonpathogenic, and those for which no definite phenotype could be assigned based on the literature were excluded from the genotype/phenotype-related outcomes.

### 2.2 Outcomes

Annualized change in eGFR was used to evaluate the rate of decline in renal function in adult patients (≥ 18 years of age) and was calculated using a four-variable MDRD [58] to enable comparison with renal outcomes in the untreated external cohort, which were calculated using the MDRD equation. [35]. An eGFR threshold of 60 mL/min/1.73 m^2^ was used to stratify patients at baseline for analysis purposes. Overt proteinuria was defined as > 0.3 g of protein in a 24-h urine collection.

Annualized left ventricular mass index (LVMI) rate of change estimates were used to evaluate progression of cardiomyopathy and were calculated using echocardiogram findings [59]. Baseline left ventricular hypertrophy (LVH) was defined as LVMI > 48 g/m^2.7^ in females and > 50 g/m^2.7^ in males.

Morbidity analyses, as time to and age at first FD-related clinical event, were performed using two clinical composite event endpoints. Composite endpoints comprised cardiac events/procedures, renal events, stroke, and death, and were described previously [20].

Survival data were based on fatality forms, adverse events forms, and exit forms.

### 2.3 Statistical analyses

Average annualized eGFR and LVMI changes were used to determine progression of renal disease and cardiomyopathy, respectively, as previously described by Beck and colleagues [20]. Rate of change estimates for eGFR and LVMI were calculated using the interaction model procedure PROCMIXED in SAS/STAT® version 9.4 (SAS Institute Inc., Cary, NC, USA). eGFR model estimations excluded values obtained following dialysis or transplantation, and serum creatinine concentration < 0.2 mg/dL or > 15 mg/dL. eGFR values < 5 mL/min/1.73 m^2^ or > 200 mL/min/1.73 m^2^ and LVMI values < 5 g/m^2.7^ or > 150 g/m^2.7^ were also excluded from the analyses.

95 % confidence intervals (CIs) were calculated for FOS data and reported for untreated comparator cohorts, where available.

Kaplan-Meier time to morbidity analyses were conducted for FOS patients from treatment start to first composite event, with censoring at the earliest of either last visit or 35 months from treatment start. Kaplan-Meier age at morbidity and age at mortality analyses were conducted for FOS patients from birth to first composite event and/or death, with censoring at last visit.

## 3 Results

### 3.1 Baseline demographics and clinical characteristics

A total of 2171 FOS patients had received agalsidase alfa treatment by August 2021 and were eligible for inclusion in these analyses (Table 1). Among those treated patients with a phenotype classification available, 79.5 % had classic FD and 20.5 % had non-classic FD (distribution of genetic variants presented in Supplementary Table 1). The treated adult population comprised 1933 patients, of whom 52.5 % were male and 47.5 % were female, and 238 treated children, of whom 68.5 % were male and 31.5 % were female. When stratified by treatment length, 1665 patients (76.7 %) had a treatment length of < 10 years, 322 patients (14.8 %) had a treatment length of ≥ 10 to < 15 years, 176 patients (8.1 %) had a treatment length of ≥ 15 to < 20 years, and 8 patients (0.4 %) had a treatment length of ≥20 years. The percentage of male patients in each treatment length group was 52.5 %, 55.6 %, 65.9 %, and 100 % in the < 10 years, ≥ 10 to < 15 years, ≥ 15 to <20 years, and ≥ 20 years groups, respectively.

**Table 1:** Baseline demographics and clinical characteristics of treated FOS patients.

Characteristic	Male (*n* = 1177)	Female (*n* = 994)	Total (*N* = 2171)
Mean age at onset of symptoms (SD), years	19.26 (17.09)	26.52 (18.19)	22.32 (17.92)
*n*	807	587	1394
Mean age at diagnosis (SD), years	30.92 (17.82)	40.17 (17.05)	35.13 (18.07)
*n*	1129	942	2071
Mean age at baseline (SD), years	37.29 (16.34)	45.58 (16.38)	41.09 (16.87)
*n*	1177	994	2171
Age group at baseline (children; adults), *n* (%)	163 (13.8); 1014 (86.2)	75 (7.5); 919 (92.5)	238 (11.0); 1933 (89.0)
Mean age at last visit (SD), years	44.78 (16.03)	52.40 (16.52)	48.27 (16.69)
*n*	1177	994	2171
Disease type (classic; non-classic), *n* (%)	288 (72.9); 107 (27.1)	290 (87.3); 42 (12.7)	578 (79.5); 149 (20.5)
Mean LVMI at baseline (SD), g/m^2.7^	58.00 (25.92)	53.01 (23.52)	55.69 (24.96)
*n*	584	502	1086
Mean eGFR at baseline (SD), mL/min/1.73 m^2^	96.44 (41.70)	94.08 (26.03)	95.35 (35.36)
*n*	882	754	1636

Baseline is defined as the most recent value within 12 months of the earliest start of agalsidase alfa.

eGFR, estimated glomerular filtration rate; FOS, Fabry Outcome Survey; LVMI, left ventricular mass index; SD, standard deviation.

Generally, treated FOS participants in this analysis developed FD symptoms as adolescents and young adults (mean age [standard deviation (SD)], years: 22.32 [17.92]). Male patients were younger at onset of symptoms, diagnosis, and treatment baseline versus females (mean [SD], years: age at symptom onset: 19.26 [17.09] vs 26.52 [18.19]; age at diagnosis: 30.92 [17.82] vs 40.17 [17.05]; age at baseline: 37.29 [16.34] vs 45.58 [16.38]). When stratified by treatment length, adult patients treated for <10 years were older at onset of symptoms, diagnosis, and treatment baseline than those treated for ≥10 years (mean [SD], years: age at symptom onset: 30.14 [19.11] vs 21.98 [17.30]; age at diagnosis: 42.59 [15.98] vs 36.03 [16.59]; age at baseline: 47.07 [14.47] vs 43.27 [14.29]. At treatment baseline, LVMI and eGFR were generally comparable between male and female patients (Table 1).

At data extraction, treated patients had received agalsidase alfa treatment for up to 20.8 years, with a median (range) accumulated time on agalsidase alfa of 5.38 (0–20.8) years. Accumulated time on treatment was similar in males and females (median [range], years: 5.51 [0.0–20.8] and 5.27 [0.0–19.5], respectively).

### 3.2 Renal impairment

#### 3.2.1 Baseline function-related clinical features

Age at symptom onset and diagnosis were later in the treated population than in the untreated comparator cohort published by Schiffmann, et al. [35] (mean [range], years: treated population: 22.32 [0.0–72.0] and 35.13 [0.0–80.0], respectively; untreated external cohort: 12.6 [0.3–56.1] and 26.0 [0–76.2], respectively). When cohorts were stratified according to eGFR and urinary protein concentration at baseline, baseline renal function characteristics and age were broadly similar within each subgroup between the FOS Evaluable Treated Renal cohort and the untreated external cohort (Supplementary Table 2).

In both cohorts, male patients with a baseline eGFR < 60 mL/min/1.73 m^2^ had the highest incidence of overt proteinuria at baseline. Within this subgroup, a lower incidence of overt proteinuria was observed in the treated population than in the untreated external cohort.

When classified according to baseline concentration of urinary protein (≥ 1.0, 0.1–< 1.0, or < 0.1 g/24 h), male and female patients with the lowest concentrations had the highest eGFR in the FOS cohort and in the untreated external cohort. Males with a urinary protein concentration of < 0.1 g/24 h had the highest eGFR overall in both cohorts.

#### 3.2.2 Renal outcomes

**Fig. 2:**
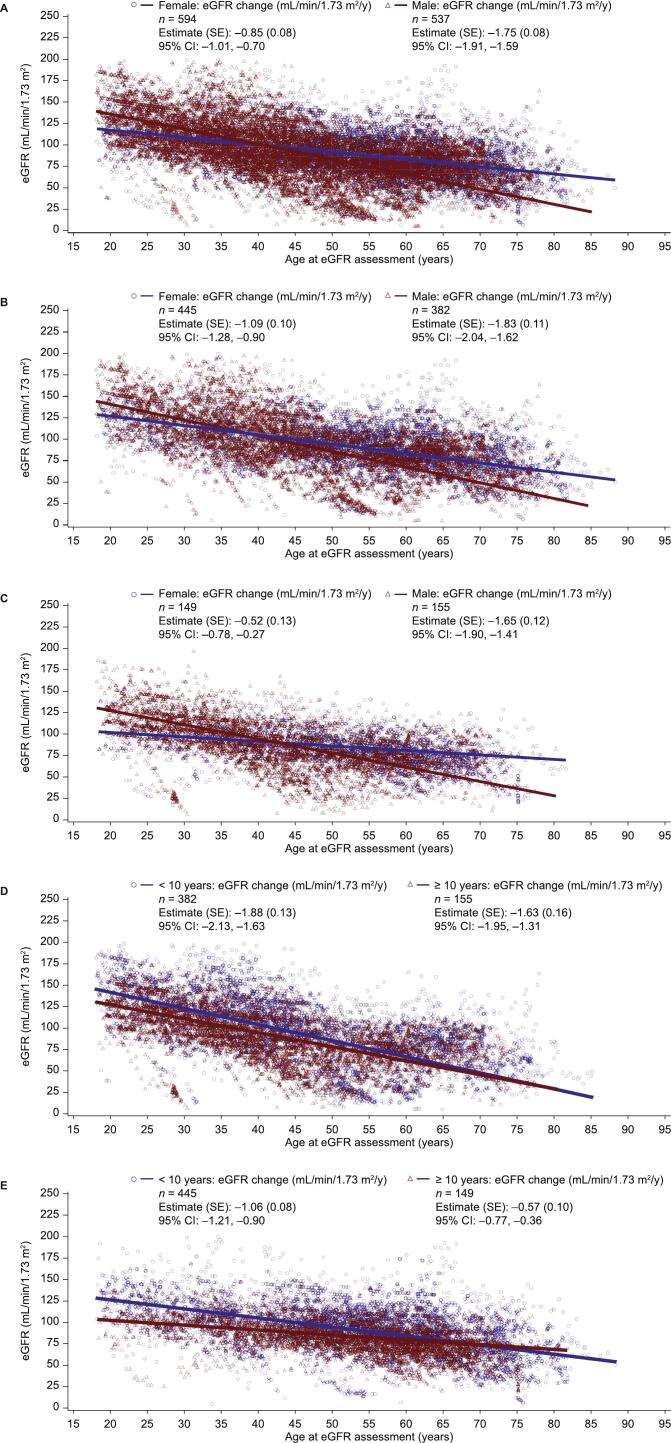
Annualized eGFR change in treated population stratified by sex and time on treatment. A) Treated patients, B) treated patients with < 10 years of treatment, C) treated patients with ≥ 10 years of treatment, D) treated males, < 10 years of treatment vs ≥ 10 years of treatment, and E) treated females, < 10 years of treatment vs ≥ 10 years of treatment. Baseline is defined as the most recent value within 12 months of the earliest start of agalsidase alfa. CI, confidence interval; eGFR, estimated glomerular filtration rate; FOS, Fabry Outcome Survey; SE, standard error; y, year.

In treated patients, the mean annual rate of eGFR decline was significantly greater in males than in females (mean [standard error of the mean, SEM] change, mL/min/1.73 m^2^/y: −1.75 [0.08] vs −0.85 [0.08]; *p* < 0.0001; Fig. 2A) and was significant within each sex subgroup (both *p* < 0.0001).

When patients were stratified by baseline eGFR (≥ 60 or < 60 mL/min/1.73 m^2^), the mean annual rate of eGFR decline in treated patients was numerically lower than the annual rate of eGFR decline in the untreated external cohorts published by Schiffmann, et al. [35], regardless of sex (Table 2). When patients were stratified by baseline urinary protein level (≥ 1.0, 0.1–< 1.0, or < 0.1 g/24 h), all treated patients had significantly less steep mean annualized eGFR change than their respective untreated external cohorts published by Schiffmann, et al. [35] (Table 2). A steeper decline in eGFR was observed in male patients with ≥ 1.0 g/24 h baseline urinary protein than in those with < 0.1 g/24 h, irrespective of treatment status.

**Table 2:** Annualized eGFR change in treated and untreated patients stratified by baseline eGFR and urinary protein levels.

		FOS Evaluable Treated Renal cohort (*N* = 1192)			Comparator untreated external cohort (*N* = 447) [35]	
Baseline eGFR category (mL/min/1.73 m^2^)	*n*	Mean annualized eGFR change (SEM), mL/min/1.73 m^2^/y	(95 % CI)	*p* value^a^	*n*	Mean annualized eGFR change (SEM), mL/min/1.73 m^2^/y
Male						
≥ 60	490	−1.68 (0.10)	−1.88, −1.48	< 0.0001	117	−3.0 (0.1)
< 60	47	−1.60 (0.34)	−2.27, −0.94	< 0.0001	28	−6.8 (1.5)
Female						
≥ 60	529	−0.78 (0.07)	−0.91, −0.65	< 0.0001	42	−0.9 (0.9)
< 60	65	−0.48 (0.19)	−0.86, −0.10	0.0125	13	−2.1 (1.6)

Baseline urinary protein level category (g/24 h)	*n*	Mean annualized eGFR change (SEM), mL/min/1.73 m^2^/y	(95 % CI)	*p* value^a^	*n*	Mean annualized eGFR change (SEM), mL/min/1.73 m^2^/y

Male						
≥ 1.0	52	−2.67 (0.34)	−3.34, −2.00	< 0.0001	22	−6.9 (1.5)
0.1–< 1.0	193	−1.78 (0.16)	−2.09, −1.46	< 0.0001	21	−3.3 (1.8)
< 0.1	78	−1.27 (0.26)	−1.78, −0.77	< 0.0001	18	−1.6 (1.5)
Female						
≥ 1.0	38	−0.88 (0.25)	−1.36, −0.39	0.0004	5	−4.6 (2.3)
0.1–< 1.0	194	−0.82 (0.11)	−1.03, −0.60	< 0.0001	17	−2.2 (2.2)
< 0.1	118	−0.83 (0.14)	−1.11, −0.56	< 0.0001	7	−0.6 (2.6)

Baseline is defined as the most recent value within 12 months of the earliest start of agalsidase alfa in FOS, or at baseline eGFR assessment in the comparator untreated population.

CI, confidence interval; eGFR, estimated glomerular filtration rate; FOS, Fabry Outcome Survey; SEM, standard error of the mean.

a This *p* value pertains to the significance of the comparison of mean annualized eGFR change to no change in eGFR.

The annualized rate of eGFR decline (mean [SEM] change, mL/min/1.73 m^2^/y) in adult patients in FOS with < 10 years of treatment was −1.42 (0.06); in those with ≥ 10 years of treatment, the rate of eGFR decline was −1.10 (0.08). When stratified by sex, male patients had a significantly faster annualized eGFR decline than their female counterparts regardless of treatment length (mean [SEM] change, mL/min/1.73 m^2^/y: < 10 years of treatment: −1.83 [0.11] vs −1.09 [0.10]; *p* < 0.0001, Fig. 2B; ≥ 10 years of treatment: −1.65 [0.12] vs −0.52 [0.13]; *p* < 0.0001, Fig. 2C). When stratified by treatment length, females with <10 years of treatment had a higher rate of eGFR decline than those treated for ≥ 10 years (*p* = 0.0002); there was no significant difference in the annualized eGFR change in males (Fig. 2D, E). The rate of eGFR decline was significant within each subgroup regardless of treatment length or sex (all *p* < 0.0001).

### 3.3 Cardiomyopathy

#### 3.3.1 Baseline function-related clinical features

Baseline cardiac-related clinical features were similar between male and female patients in both the FOS Evaluable Treated Cardiac cohort and the comparator untreated population published by Kampmann, et al. [34] (Supplementary Table 3). In both treated and untreated patients, males were younger than females at baseline (mean [SD], years: treated patients: 37.46 [16.89] vs 46.51 [16.70]; untreated external cohort: 33.8 [12.2] vs 38.0 [18.3]). Baseline LVMI was similar in the treated cohort and the untreated external cohort (mean [SD], g/m^2.7^: males: 55.47 [24.55] vs 56.8 [27.2]; females: 54.27 [23.73] vs 48.2 [27.2]).

#### 3.3.2 Cardiac outcomes

Treated and untreated patients (published by Kampmann, et al. [34]) had positive mean annualized LVMI changes, regardless of sex. The yearly change in LVMI was significantly greater in the untreated external cohort than in the treated patients (*p* < 0.0001) (Table 3). Adult FOS patients with < 10 years of treatment had similar rates of annual LVMI change to those with ≥ 10 years of treatment (change [SEM], g/m^2.7^/y: 0.85 [0.05], *p* < 0.0001 vs 0.75 [0.06], *p* < 0.0001). When stratified by sex, annualized LVMI changes were similar between male and female patients who had received treatment for < 10 years (0.89 [0.07], *p* < 0.0001 vs 0.78 [0.07], *p* < 0.0001; *p* = 0.2745 for subgroup comparison). Similarly, in patients with ≥ 10 years of treatment, there was no statistical difference in annualized LVMI change between males and females (0.81 [0.10], *p* < 0.0001 vs 0.67 [0.10], *p <* 0.0001; *p* = 0.3510 for subgroup comparison).

**Table 3:** Annualized LVMI change in treated and untreated patients stratified by sex and presence/absence of LVH at baseline.

	FOS Evaluable Treated Cardiac cohort (*N* = 724)				Comparator untreated external cohort (*N* = 78) [34]	
Baseline LVH status	*n*	Mean annualized LVMI change (SEM), g/m^2.7^/y	95 % CI	*p* value^a^	*n*	Mean annualized LVMI change (SEM), g/m^2.7^/y
Male						
Total	345	0.67 (0.05)	0.58, 0.76	< 0.0001	39	4.07 (1.03)
LVH	152	0.77 (0.06)	0.65, 0.88	< 0.0001	18	6.59 (8.5)
No LVH	193	0.32 (0.05)	0.22, 0.41	< 0.0001	−b	−b

Female						
Total	343	0.32 (0.04)	0.25, 0.39	< 0.0001	39	2.31 (0.81)
LVH	181	0.57 (0.08)	0.42, 0.72	< 0.0001	15	3.77 (7.7)
No LVH	162	0.33 (0.05)	0.23, 0.44	< 0.0001	−b	−b

Baseline is defined as the most recent value within 12 months of the earliest start of agalsidase alfa in FOS, or at baseline LVMI assessment in the comparator untreated population.

LVH is defined as LVMI ≥ 51 g/m^2.7^ for males and LVMI ≥ 48 g/m^2.7^ for females.

CI, confidence interval; FOS, Fabry Outcome Survey; LVH, left ventricular hypertrophy; LVMI, left ventricular mass indexed to height; SEM, standard error of the mean.

a This *p* value pertains to the significance of the comparison of mean annualized LVMI change to no change in LVMI.

b These data are not available.

In patients with LVH at baseline, treated and untreated patients had positive annualized LVMI changes (Table 3). The annualized LVMI changes of treated patients with LVH were comparable to those of treated patients with no LVH, regardless of sex (Table 3).

### 3.4 Morbidity analyses

#### 3.4.1 Baseline function-related clinical features

There was a lower proportion of male patients in the Evaluable Treated Morbidity cohort than in the untreated external cohorts published by Banikazemi, et al. [33] and Schiffmann, et al. [35] (46.6 % vs 87.0 % and 62.4 %, respectively; Supplementary Table 4). Mean baseline urinary protein concentrations and eGFR values were comparable between the Evaluable Treated Morbidity cohort and the untreated external cohort [33]. Baseline characteristics for the untreated external cohort reported by Schiffmann, et al. [35] were compared in the previous section (Supplementary Table 2).

#### 3.4.2 Morbidity outcomes

**Fig. 3:**
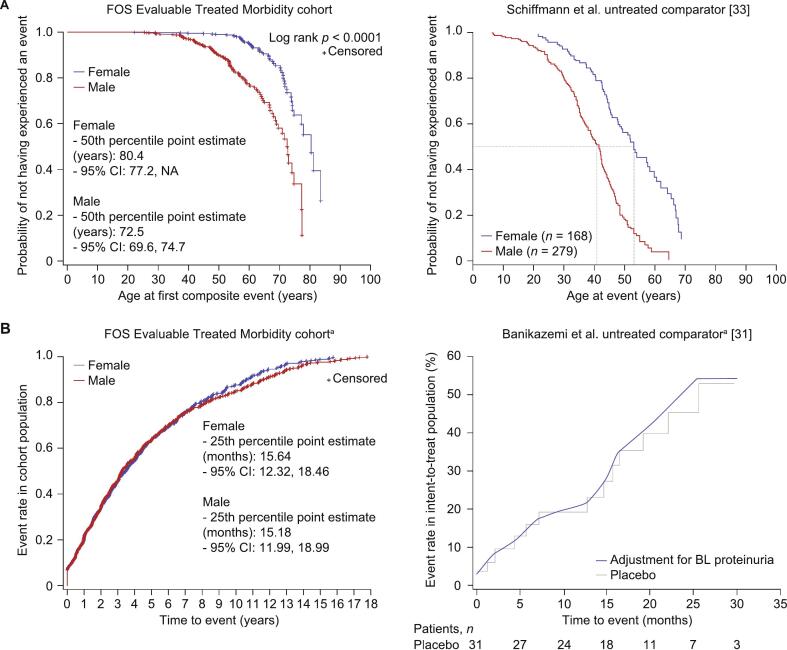
Morbidity outcomes in the treated population stratified by sex. A) Age at first renal, cardiac, or stroke event, or death, in treated patients, and B) time to first renal, cardiac, or stroke event, or death, in treated patients. CI, confidence interval; FOS, Fabry Outcome Survey. ^a^ Owing to the wide differences in range of event rate data between the FOS population and the published comparator, and to ensure that both data sets are being represented appropriately, the axis ranges used in the graphs of panel B differ from each other.

In the treated group, male patients were generally younger than female patients at their first composite event (mean age [SD], years: males: 40.23 [15.82] vs females: 49.14 [16.10]; Fig. 3A) and treated patients were older than those from the untreated external cohort. In the untreated external cohort, approximate age at first composite event was < 10 years for males and > 20 and < 25 years for females [35]. The age at which half of the patients had experienced their first composite event was greater in the treated population than in the untreated external cohort published by Schiffmann, et al. [35] (estimate, years: males: 51.7 vs 41.0; females: 60.8 vs 53.0). Male and female treated patients had a similar time from treatment start to first composite event (median [95 % CI], months: 38.9 [36.1, 44.6] vs 41.6 [36.5, 47.1]; Fig. 3B).

After 24 months of treatment, the probability of a composite morbidity event was approximately 34 % in the FOS Morbidity cohort (46.6 % males) and approximately 45 % in the untreated external cohort (87.0 % males) published by Banikazemi, et al. [33].

### 3.5 Survival analysis

#### 3.5.1 Baseline function-related clinical features

The Evaluable Treated cohort consisted of 1999 patients; 172 patients from the All Treated cohort were excluded as they had undergone dialysis, kidney transplantation and/or heart transplantation before starting FD treatment). The Evaluable Treated cohort was generally older than the untreated external cohort published by Schiffmann, et al. [35] at symptom onset, diagnosis, and data extraction (Supplementary Table 5 A).

Treated patients who died during FOS had a similar age at onset of symptoms but were generally older at diagnosis, at initiation of agalsidase alfa treatment, and at time of last visit than those who did not die (mean age [SD], years: symptom onset: 23.73 [18.69] vs 22.15 [17.83]; diagnosis: 41.65 [18.73] vs 34.44 [17.87]; baseline: 52.71 [12.59] vs 39.89 [16.80]; last visit: 59.99 [12.04] vs 47.06 [16.64]; Supplementary Table 5B). Most of the patients who died (99 %; 201/203) were adults at treatment start and had a higher prevalence of LVH and eGFR < 60 mL/min/1.73 m^2^ than those who survived (LVH at baseline, n [%]: 100 [87.7] vs 454 [46.7]; eGFR < 60 mL/min/1.73 m^2^ at baseline, n [%]: 74 [43.5] vs 169 [11.5]).

#### 3.5.2 Survival outcomes

**Fig. 4:**
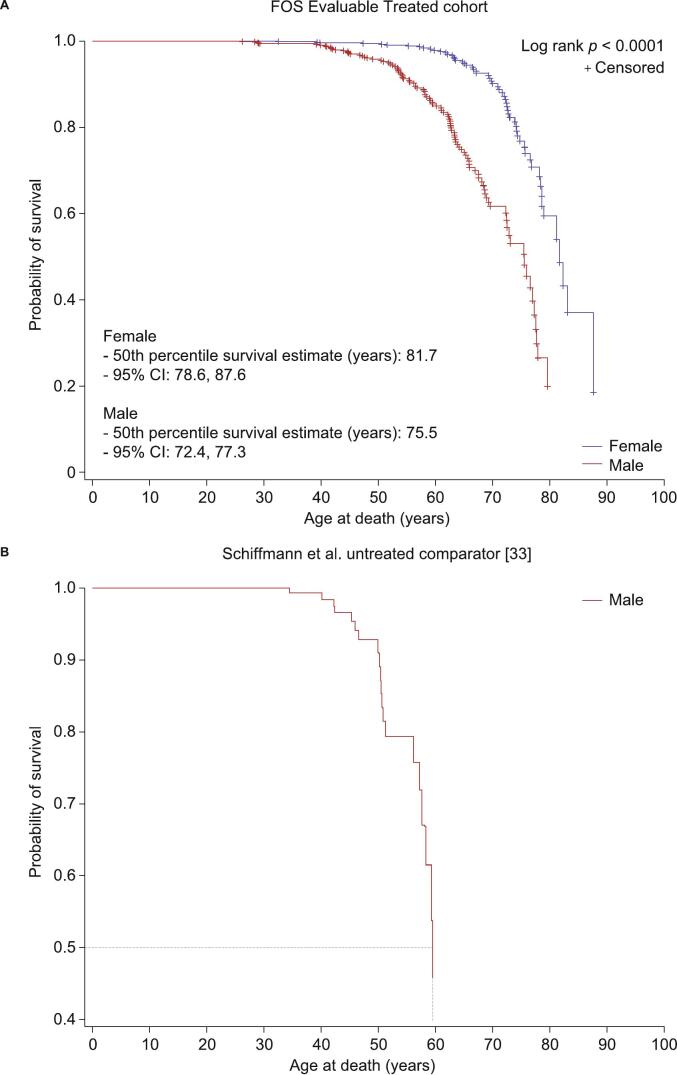
Survival over time in treated and untreated patients stratified by sex. A) Treated FOS patients stratified by sex, and B) the untreated external cohort published by Schiffmann et al. [35] CI, confidence interval; FOS, Fabry Outcome Survey.

Treated patients were older at death than untreated patients from the external cohort (mean [range], years: 61.7 [26.2, 87.6] vs 50.3 [34.5, 70.1]) [35]. In treated patients stratified by sex, males had a lower probability of survival over time than females (Fig. 4A). The age at which 50 % of male patients were still alive was higher in treated patients than in the untreated external cohort (estimate, years: 75.5 vs 60.0; Fig. 4B) [35].

The most common known causes of death in treated patients were related to cardiovascular events (34.7 %, n/*N* = 70/202), followed by unknown causes (12.4 %, 25/202), cerebrovascular events (11.9 %, 24/202), and respiratory issues (9.9 %, 20/202). The most common cardiovascular causes of death were heart failure (40.0 %, n/*N* = 28/70) and arrhythmias (21.4 %, n/*N* = 15/70).

### 3.6 Genetic and phenotypic analysis

#### 3.6.1 Baseline demographics and clinical characteristics

The collection of genotype data in FOS was optional and dependent on patient consent. In this cohort, genotype data was available for 33 % (727/2171) of patients. Treated patients with classic FD were generally younger than those with non-classic FD at symptom onset, diagnosis, start of agalsidase alfa treatment, and last visit (mean [SD], years: symptom onset: 17.88 [14.73] vs 42.36 [17.99]; diagnosis: 31.27 [16.99] vs 49.64 [16.38]).

Patients with classic FD (mean [SD] age at baseline: 37.84 [16.12] years) had higher eGFR at baseline than those with non-classic FD (mean [SD] age at baseline: 52.88 [14.59] years). eGFR and LVMI at baseline were similar for male and female patients with classic FD (Supplementary Table 6).

#### 3.6.2 Clinical outcomes stratified by FD phenotype

**Fig. 5:**
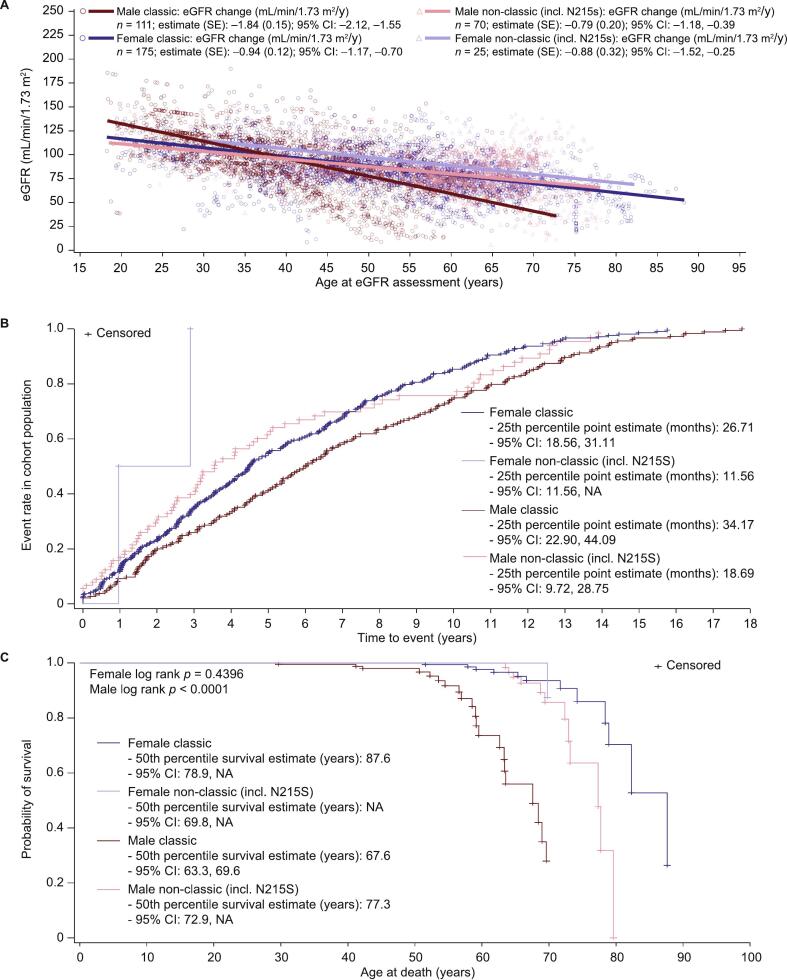
Outcomes for treated FOS patients with classic or non-classic FD. A) Annualized eGFR change, B) time to first renal, cardiac, or stroke event, or death, and C) survival curves. Data points represent individual patient data. CI, confidence interval; eGFR, estimated glomerular filtration rate; FD, Fabry disease; FOS, Fabry Outcome Survey; NA, not available; SE, standard error; y, year.

In male patients, the eGFR annual rate of decline was significantly greater in patients with classic FD than in those with non-classic FD; this difference was not seen in female patients (males: *p* < 0.0001; females: *p* = 0.875; Fig. 5A). The annualized eGFR decline was significant within each FD phenotype regardless of sex (change [SEM], mL/min/1.73 m^2^/y: classic FD, males: −1.84 [0.15; *p* < 0.0001], females: −0.94 [0.12; *p* < 0.0001]; non-classic FD, males: −0.79 [0.20; *p* < 0.0001], females: −0.88 [0.32; *p* = 0.007]; Fig. 4A).

LVMI annual rates of change were not significantly different in male or female patients when stratified by disease phenotype (males: *p* = 0.0847; females: *p* = 0.425). However, all subgroups had a significant positive annualized change in LVMI (change [SEM]: classic FD, males: 0.62 [0.08; *p* < 0.0001], females: 0.57 [0.12; *p* < 0.0001]; non-classic FD, males: 0.83 [0.10; *p* < 0.0001], females: 0.84 [0.32; *p* = 0.009]).

Treated patients with classic FD had a longer time from treatment initiation to first renal, cardiac, or cerebrovascular event, or death than those with non-classic FD, regardless of sex (median [95 % CI] time to first event, months: males: 72.15 [62.36, 82.60] vs 42.58 [34.73, 59.56]; females: 53.95 [47.41, 63.97] vs 23.18 [11.56, not available, NA]; Fig. 5B).

Treated patients with classic FD had a younger mean age at death than those with non-classic FD (mean [SD], years: 47.36 [16.21] vs 59.51 [14.02]; Fig. 5C). When stratified by sex, male patients with classic FD had a younger age of 50 % survival than those with non-classic FD or female patients with classic FD (mean [95 % CI], years: males with classic FD: 67.6 [63.3, 69.6]; males with non-classic FD: 77.3 [72.9, NA]; females with classic FD: 87.6 [78.9, NA]. There were not enough data available to calculate the 50 % survival of female patients with non-classic FD.

## 4 Discussion

This study reports real-world characteristics and clinical outcomes for patients with FD who are enrolled in the FOS registry and have been treated with agalsidase alfa for up to 20.8 years. When these patients were stratified by sex, males had a greater decline in eGFR, were younger at the time of their first composite event and had a lower probability of survival over time than females. Generally, treated FOS patients had lower rates of eGFR and LVMI progression, and were older at first composite event and at death than their respective comparator untreated populations (using external cohorts from the literature [[33], [34], [35]]). Mortality findings were particularly notable, with treated patients having an average survival of over 10 years longer than untreated patients from published cohorts [33,34]. Overall, these findings indicate a positive impact of agalsidase alfa treatment on renal outcomes, as well as a stabilization or slowing of cardiomyopathy progression and delayed morbidity and mortality in treated patients when compared with untreated comparator cohorts.

Sex and baseline disease severity appeared to have an effect on clinical outcomes in several subgroups of patients. In our analyses, female patients generally had more stable annual eGFR rates and a higher probability of overall survival than male patients. These differences in clinical outcomes that were dependent on sex, especially in patients receiving agalsidase alfa, were expected. It is widely recognized that FD generally presents more severely in male patients than in female patients, particularly classic FD. Indeed, until the 21st century, female patients with FD were thought to be asymptomatic carriers or to only develop mild symptoms. However, it is now known that female patients with FD can develop substantial manifestations of the disease and have an increased risk of premature death when they remain untreated [36,[60], [61], [62], [63]].

When stratified by baseline urinary protein concentration, patients with the highest concentrations (≥ 1.0 g/24 h) had the greatest change in annualized eGFR over time regardless of sex and treatment status. Indeed, proteinuria and increased serum creatinine have been previously reported as risk factors for rapid eGFR decline in patients with chronic kidney disease [33,[64], [65], [66]]. Nonetheless, patients with FD who were treated with agalsidase alfa had lower annualized eGFR changes over time than untreated patients. Since proteinuria is a sign of kidney damage and renal impairment is progressive in FD, prompt treatment may be beneficial to patients with FD in order to prevent further kidney damage caused by the disease. Furthermore, patients who had been treated for ≥10 years had less steep annualized eGFR changes than those who had been treated for <10 years. While treatment with agalsidase alfa for ≥10 years has been shown to promote clinical stability in patients with FD who had normal kidney function at treatment initiation, when compared with untreated patients with FD [19], our analyses suggest that long-term treatment with agalsidase alfa may be beneficial to patients with known *GLA* gene pathogenic variants, regardless of their clinical characteristics at treatment start.

There is broad phenotypic variability in FD, and more than 900 variants of the *GLA* gene have been identified [40]. Males with classic FD often have very low or no residual enzyme activity, and generally develop symptoms at a younger age and have a more severe disease course than patients with non-classic FD [39,67]. This was also the case in our analyses: patients with classic FD developed FD-related symptoms at a younger age than those with non-classic FD. In our cohort, those with classic disease were on average 15 years younger and had higher eGFR at treatment initiation than those with non-classic FD. Notably, males with non-classic disease had a higher baseline LVMI than males with classic disease, but the difference in LVMI was not seen among females. Moreover, male patients with classic FD had a lower overall survival rate than those with non-classic FD, further indicating that classic FD affects patients at a younger age and more aggressively than non-classic FD.

This study was a continuation of the analyses reported by Beck and colleagues in 2015 [20]; while they reported clinical data of patients with agalsidase alfa treatment of approximately 5 years, treated patients in our data set had a mean exposure time of approximately 7 years and included a subgroup of patients treated for 10 years or more. Moreover, the cohort reported by Beck and colleagues [20] included 677 patients, while we report data from 2171 patients. The effect of agalsidase alfa on clinical outcomes reported by Beck et al. [20] was comparable to our results, demonstrating the potential benefits of treatment with agalsidase alfa in a wide population of patients with FD.

The protective effects of agalsidase alfa treatment on the progression of renal impairment, cardiomyopathy, and mortality in FD reported here show similar trends to previously published studies [[19], [20], [21], [22], [23], [24], [25], [26], [27], [28]]. However, to our knowledge, the current set of analyses represents the longest overall reported experience of treatment with agalsidase alfa and makes a valuable addition to our understanding of outcomes with ERT and the impact of different patient characteristics – including sex, treatment length, baseline disease severity, and phenotype/genotype – on treatment.

Some limitations of the current set of analyses have to be considered in order to properly evaluate these results in a general context. Firstly, this was a retrospective analysis of data extracted from the FOS disease registry and, as such, it is difficult to ensure that the data capture was comprehensive. For instance, renal and cardiac outcomes are affected by risk factors including blood pressure and smoking. However, the incidence of risk factors such as smoking, blood pressure, dyslipidemia, and use of angiotensin receptor blockers or angiotensin-converting enzyme inhibitors was not consistently captured in this registry or in the comparator untreated populations. Additionally, patients with severe disease symptoms or who are receiving treatment are more likely to be enrolled in a disease registry; therefore, there is a possibility of patient enrollment bias. Findings from patient registry data may not be generalizable to all patients with FD, although the high number of patients included in these analyses may improve the accuracy of our results and better reflect real-world outcomes. It is important to note that comparison with published data from untreated patients was limited by the fact that the populations were from different data sets, and it was not possible to directly match treated patients from FOS with these comparator data sets, owing to differences in baseline characteristics, endpoint definitions, and study duration. Untreated patients in FOS may have been considered the ideal comparators in terms of comparable data collection methodology; however, patient numbers were relatively small. Additionally, disease progression in the treated cohort is affected by time of treatment initiation in relation to disease severity, which might have influenced outcomes, especially considering that the current FOS population maybe be more heterogeneous than in the early days when it was mostly males with a classic severe phenotype [33,35]. Another limitation to consider is that the FOS patients increasingly benefit from better adjunctive care over time, especially compared with those patients in the untreated comparator groups, for some of whom data were retrospectively collected up to 15 years earlier.

## 5 Conclusions

These results indicate that treatment with agalsidase alfa slowed renal deterioration and progression of cardiomyopathy, and delayed morbidity and death in patients with FD who were enrolled in FOS. As such, long-term treatment with agalsidase alfa may provide renal, cardiac, and overall survival protection in patients with FD. The data support and extend previous findings on the efficacy of agalsidase alfa in a large patient population over a long-term period, in different subgroups of patients treated for up to 20.8 years, making a valuable contribution to our understanding of outcomes following treatment with ERT.

## Abbreviations

**Table:** 

α-Gal A	alpha-Galactosidase A
CI	Confidence interval
dbFGP	International Fabry Disease Genotype-Phenotype Database
eGFR	Estimated glomerular filtration rate
ERT	Enzyme replacement therapy
FD	Fabry disease
FOS	Fabry Outcome Survey
Gb_3_	Globotriaosylceramide
*GLA*	Galactosidase alpha
LVH	Left ventricular hypertrophy
LVMI	Left ventricular mass index
MDRD	Modification of Diet in Renal Disease
NA	not available
SD	Standard deviation
SE	Standard error
SEM	Standard error of the mean
y	year

## Funding

FOS and this analysis were funded by Takeda Pharmaceuticals International AG, which also assisted in analyzing the data and preparing the manuscript. Under the direction of the authors, medical writing support was provided by Ester Baixauli PhD of Oxford PharmaGenesis and funded by Takeda Development Center Americas, Inc. The final decision to submit the manuscript for publication was made by the authors.

## CRediT authorship contribution statement

**Uma Ramaswami:** Writing – review & editing, Writing – original draft, Supervision, Resources, Methodology, Investigation, Data curation, Conceptualization. **Guillem Pintos-Morell:** Writing – review & editing, Writing – original draft, Supervision, Resources, Methodology, Investigation, Data curation, Conceptualization. **Christoph Kampmann:** Writing – review & editing, Writing – original draft, Supervision, Resources, Methodology, Investigation, Data curation, Conceptualization. **Kathleen Nicholls:** Writing – review & editing, Writing – original draft, Supervision, Resources, Methodology, Investigation, Data curation, Conceptualization. **Dau-Ming Niu:** Writing – review & editing, Writing – original draft, Supervision, Resources, Methodology, Investigation, Data curation, Conceptualization. **Ricardo Reisin:** Writing – review & editing, Writing – original draft, Supervision, Resources, Methodology, Investigation, Data curation, Conceptualization. **Michael L. West:** Writing – review & editing, Writing – original draft, Supervision, Resources, Methodology, Investigation, Data curation, Conceptualization. **Christina Anagnostopoulou:** Writing – review & editing, Writing – original draft, Supervision, Software, Resources, Project administration, Methodology, Investigation, Funding acquisition, Data curation, Conceptualization. **Jaco Botha:** Writing – review & editing, Writing – original draft, Visualization, Validation, Supervision, Software, Resources, Project administration, Methodology, Investigation, Funding acquisition, Formal analysis, Data curation, Conceptualization. **Dalia Jazukeviciene:** Writing – review & editing, Writing – original draft, Supervision, Software, Resources, Project administration, Methodology, Investigation, Funding acquisition, Data curation, Conceptualization. **Jörn Schenk:** Writing – review & editing, Writing – original draft, Supervision, Software, Resources, Project administration, Methodology, Investigation, Funding acquisition, Data curation, Conceptualization. **Derralynn A. Hughes:** Writing – review & editing, Writing – original draft, Supervision, Resources, Methodology, Investigation, Data curation, Conceptualization. **Roberto Giugliani:** Writing – review & editing, Writing – original draft, Supervision, Resources, Methodology, Investigation, Data curation, Conceptualization.

## Declaration of competing interest

UR reports honoraria for speaking and/or advisory boards from Amicus Therapeutics, Sanofi Genzyme, and Takeda, and research grants from Amicus Therapeutics, Intrabio, and Takeda. She is a member of the FOS Steering Committee. GP-M reports honoraria from Alexion, Amicus Therapeutics, BioMarin, Chiesi, Kyowa-Kirin, Lucane, Sanofi, and Takeda, and unrestricted grants from Sanofi and Takeda to the Vall d'Hebron 10.13039/100005930Research Foundation for funding research on rare diseases. He is a member of the FOS Steering Committee. CK reports honoraria for speaking and/or advisory boards from Amicus Therapeutics, BioMarin, Gore, and Takeda. He is a member of the FOS Steering Committee. KN reports honoraria from Amicus Therapeutics, Sanofi Genzyme, and Takeda. She is a member of the FOS Steering Committee. D-MN reports honoraria and speaker fees from Sanofi Genzyme and Takeda, and research grants from BioMarin, Sanofi Genzyme, and Takeda. He is a member of the FOS Steering Committee. RR reports honoraria, speaker fees, and consulting fees from Amicus Therapeutics, CSL Behring, Gador, Novartis, Sanofi Genzyme, and Takeda. He is a member of the FOS Steering Committee. MLW reports grants, personal fees, and travel support from 10.13039/100006396Alexion, Alnylam, 10.13039/100015362Amicus Therapeutics, Chiesi, Idorsia, Moderna, Protalix, 10.13039/100013995Sanofi Genzyme, and Takeda. He is a member of the FOS Steering Committee. CA is a former employee of Takeda Pharmaceuticals International AG and a current employee of Medison Pharma, Switzerland. JB is an employee of Takeda Pharmaceuticals International AG and is a stockholder of Takeda Pharmaceuticals Company Limited. DJ is an employee of Takeda Pharmaceuticals International AG and is a stockholder of Takeda Pharmaceuticals Company Limited. JS is a former employee of Takeda Pharmaceuticals International AG and a current employee of Kalvista Pharmaceuticals, Switzerland. DAH reports honoraria from Amicus Therapeutics, Chiesi, Freeline Therapeutics, Idorsia, Protalix, Sanofi Genzyme, and Takeda. She is a member of the FOS Steering Committee. RG reports honoraria, consulting fees, speaker fees, research funding, and/or travel reimbursement from Alexion, Allievex, Alnylam, Amicus Therapeutics, Astellas, Azafaros, BioMarin, Chiesi, JCR, Lysogene, Novartis, Paradigm, PassageBio, Pfizer, Praxis, PTC, Regenxbio, Sanofi, Takeda, and Ultragenyx. He is a member and the current chair of the FOS Steering Committee.

## Data Availability

The datasets, including the redacted study protocol, redacted statistical analysis plan, and individual participants data supporting the results reported in this article, will be made available within three months from initial request, to researchers who provide a methodologically sound proposal. The data will be provided after its de-identification, in compliance with applicable privacy laws, data protection, and requirements for consent and anonymization.

## Appendix A Supplementary data

Supplementary material Image 1
