# Supplementary material for: Two decades of experience of the Fabry Outcome Survey provides further confirmation of the long-term effectiveness of agalsidase alfa enzyme replacement therapy
Source: Mol Genet Metab Rep. 2025 Apr 11;43:101215. doi: 10.1016/j.ymgmr.2025.101215 (PMC12018052; doi:10.1016/j.ymgmr.2025.101215)
Supplement: Supplementary file 1 — Supplementary material [file mmc1.pdf]

Supplementary materials

Two decades of experience of the Fabry Outcome Survey provides further confirmation of the long-term effectiveness of agalsidase alfa enzyme replacement therapy

Uma Ramaswami,[a] Guillem Pintos-Morell,[b] Christoph Kampmann,[c] Kathleen Nicholls,[d] Dau-Ming Niu,[e] Ricardo Reisin,[f] Michael L. West,[g] Christina Anagnostopoulou,[h,1] Jaco Botha,[h,*] Dalia Jazukeviciene,[h] Jörn Schenk,[h,2] Derralynn A. Hughes,[a] Roberto Giugliani[i]

[a]Royal Free London NHS Foundation Trust, University College London, Pond Street, London, NW3 2QG, UK

[b]Vall d'Hebron Institute of Research (VHIR), Vall d'Hebron Barcelona Hospital Campus, Passeig Vall d'Hebron 119-129, 08035 Barcelona, Spain

[c]Johannes Gutenberg School of Medicine, University of Mainz, Saarstraße 21, 55122 Mainz, Germany

[d]The Royal Melbourne Hospital and the University of Melbourne, 300 Grattan Street, Parkville, VIC 3052, Australia

[e]Taipei Veterans General Hospital, No. 201, Section 2, Shih-Pai Road, Taipei 112, Taiwan

[f]Hospital Británico de Buenos Aires, Perdriel 74, C1280AEB Cdad., Buenos Aires, Argentina

[g]Department of Medicine, Dalhousie University, 5849 University Avenue, Halifax, NS, B3H 4R2, Canada

[h]Takeda Pharmaceuticals International AG, Thurgauerstrasse 130, 8152 Glattpark (Opfikon), Zurich, Switzerland

[i]Department of Genetics, UFRGS, Medical Genetics Service, HCPA, INAGEMP, Dasa Genomica and Casa dos Raros, Rua Sao Manoel 730, Porto Alegre, RS 90610-261, Brazil)

[1]Affiliation at the time of the analysis was Takeda Pharmaceuticals International AG, Zurich, Switzerland; current affiliation is Medison Pharma, Zug, Switzerland

[2]Affiliation at the time of the analysis was Takeda Pharmaceuticals International AG, Zurich, Switzerland; current affiliation is KalVista Pharmaceuticals, Zug, Switzerland

SUPPLEMENTARY TABLES

Supplementary Table 1 Treated FOS patients stratified by FD-related genetic variant, disease type, age group, and sex.

A) Patients with non-classic FD

**Genetic variant**	**Adult patients**		**Pediatric patients**	
	**Male (*n* = 104)**	**Female (*n* = 40)**	**Male (*n* = 3)**	**Female (*n* = 2)**
D231N	0 (0.0)	1 (2.5)	–	–
inversion	0 (0.0)	1 (2.5)	–	–
M296I	0 (0.0)	2 (5.0)	–	–
Q280H	0 (0.0)	1 (2.5)	–	–
Y184N	0 (0.0)	1 (2.5)	–	–
deletion	1 (1.0)	0 (0.0)	–	–
I91T	1 (1.0)	1 (2.5)	–	–
L129P	–	–	0 (0.0)	1 (50.0)
L300P	1 (1.0)	0 (0.0)	–	–
N139S	1 (1.0)	0 (0.0)	–	–
R112H	1 (1.0)	3 (7.5)	0 (0.0)	1 (50.0)
R363H	1 (1.0)	0 (0.0)	–	–
P205S	2 (1.9)	0 (0.0)	–	–
S238N	2 (1.9)	0 (0.0)	–	–
N215S	27 (26.0)	9 (22.5)	2 (66.7)	0 (0.0)
R301Q	5 (4.8)	4 (10.0)	1 (33.3)	0 (0.0)
IVS4+919G>A	58 (55.8)	13 (32.5)	–	–
F113L	4 (3.8)	4 (10.0)	–	–

B) Patients with classic FD

Genetic variant	Adult patients		Pediatric patients	
	**Male (*n* = 233)**	**Female (*n* = 266)**	**Male (*n* = 55)**	**Female (*n* = 24)**
A143P	14 (6.0)	21 (7.9)	2 (3.6)	0 (0.0)
A156D	0 (0.0)	1 (0.4)	–	–
A156T	0 (0.0)	1 (0.4)	–	–
A285D	2 (0.9)	2 (0.8)	0 (0.0)	1 (4.2)
A288D	1 (0.4)	1 (0.4)	–	–
A31V	1 (0.4)	2 (0.8)	–	–
A348P	0 (0.0)	1 (0.4)	–	–
C142R	1 (0.4)	0 (0.0)	–	–
C172Y	2 (0.9)	1 (0.4)	1 (1.8)	0 (0.0)
C223G	1 (0.4)	0 (0.0)	0 (0.0)	1 (4.2)
C378R	1 (0.4)	0 (0.0)	–	–
C52Y	0 (0.0)	1 (0.4)	–	–
C56S	1 (0.4)	0 (0.0)	–	–
C56Y	4 (1.7)	0 (0.0)	–	–
D244N	1 (0.4)	0 (0.0)	–	–
D92G	1 (0.4)	1 (0.4)	–	–
D93G	0 (0.0)	1 (0.4)	–	–
D93N	–	–	1 (1.8)	0 (0.0)
D93Y	–	–	1 (1.8)	0 (0.0)
deletion	36 (15.5)	50 (18.8)	15 (27.3)	8 (33.3)
del+ins	1 (0.4)	0 (0.0)	–	–
duplication	1 (0.4)	6 (2.3)	1 (1.8)	0 (0.0)
E338K	0 (0.0)	2 (0.8)	–	–
E48K	0 (0.0)	1 (0.4)	–	–
E48X	1 (0.4)	0 (0.0)	–	–
E66K	2 (0.9)	1 (0.4)	2 (3.6)	0 (0.0)
G128E	1 (0.4)	0 (0.0)	–	–
G132R	0 (0.0)	2 (0.8)	1 (1.8)	0 (0.0)
G138R	0 (0.0)	2 (0.8)	–	–
G183S	2 (0.9)	3 (1.1)	–	–
G261N	1 (0.4)	0 (0.0)	–	–
G261V	1 (0.4)	3 (1.1)	–	–
G325S	2 (0.9)	1 (0.4)	–	–
G328R	2 (0.9)	4 (1.5)	–	–
G360C	1 (0.4)	0 (0.0)	–	–
G361R	1 (0.4)	1 (0.4)	–	–
G361X	0 (0.0)	2 (0.8)	2 (3.6)	0 (0.0)
G373D	1 (0.4)	1 (0.4)	–	–
G411D	1 (0.4)	1 (0.4)	–	–
H46R	2 (0.9)	2 (0.8)	1 (1.8)	0 (0.0)
H46Y	0 (0.0)	1 (0.4)	–	–
I317T	4 (1.7)	0 (0.0)	–	–
I407K	1 (0.4)	0 (0.0)	–	–

Genetic variant	Adult patients		Pediatric patients	
	**Male (*n* = 233)**	**Female (*n* = 266)**	**Male (*n* = 55)**	**Female (*n* = 24)**
insertion	10 (4.3)	8 (3.0)	3 (5.5)	0 (0.0)
inversion	1 (0.4)	2 (0.8)	1 (1.8)	0 (0.0)
Intron 3	0 (0.0)	1 (0.4)	–	–
IVS2+1	1 (0.4)	0 (0.0)	–	–
IVS2+1G>A	1 (0.4)	1 (0.4)	1 (1.8)	0 (0.0)
IVS2+1G>T	1 (0.4)	1 (0.4)	–	–
IVS2A>G	2 (0.9)	1 (0.4)	–	–
IVS3+1G>A	1 (0.4)	0 (0.0)	–	–
IVS3-1G>A	0 (0.0)	1 (0.4)	–	–
IVS3-1G>C	2 (0.9)	0 (0.0)	–	–
IVS4+1G>A	0 (0.0)	1 (0.4)	–	–
IVS4+5G>T	1 (0.4)	0 (0.0)	0 (0.0)	1 (4.2)
IVS4+919G>A	1 (0.4)	0 (0.0)	–	–
IVS5+3A>G	0 (0.0)	2 (0.8)	–	–
K185X	0 (0.0)	1 (0.4)	–	–
L106F	0 (0.0)	1 (0.4)	–	–
L131P	3 (1.3)	0 (0.0)	–	–
L191P	1 (0.4)	0 (0.0)	1 (1.8)	0 (0.0)
L268S	0 (0.0)	1 (0.4)	1 (1.8)	0 (0.0)
L268X	1 (0.4)	0 (0.0)	–	–
L344P	1 (0.4)	0 (0.0)	–	–
L414S	0 (0.0)	1 (0.4)	–	–
L415P	1 (0.4)	2 (0.8)	–	–
L68F	1 (0.4)	0 (0.0)	–	–
L89P	1 (0.4)	0 (0.0)	–	–
M187R	2 (0.9)	2 (0.8)	–	–
M1K	–	–	1 (1.8)	0 (0.0)
M1T	0 (0.0)	1 (0.4)	–	–
M284T	2 (0.9)	0 (0.0)	–	–
M42T	1 (0.4)	1 (0.4)	–	–
M42V	2 (0.9)	0 (0.0)	–	–
N224D	1 (0.4)	0 (0.0)	–	–
N249K	0 (0.0)	2 (0.8)	–	–
N272S	0 (0.0)	3 (1.1)	1 (1.8)	0 (0.0)
N278K	0 (0.0)	1 (0.4)	–	–
N298H	0 (0.0)	1 (0.4)	0 (0.0)	2 (8.3)
N320I	1 (0.4)	0 (0.0)	–	–
N34S	1 (0.4)	1 (0.4)	–	–
other	0 (0.0)	1 (0.4)	–	–
p.(ASN139Lysfs*2)	0 (0.0)	1 (0.4)	–	–
P205S	1 (0.4)	0 (0.0)	–	–
P205T	1 (0.4)	3 (1.1)	–	–
P259R	0 (0.0)	2 (0.8)	–	–
P40L	1 (0.4)	0 (0.0)	–	–
Q107X	0 (0.0)	5 (1.9)	1 (1.8)	0 (0.0)
Q157X	2 (0.9)	0 (0.0)	1 (1.8)	0 (0.0)

Genetic variant	Adult patients		Pediatric patients	
	Male (*n* = 233)	Female (*n* = 266)	Male (*n* = 55)	Female (*n* = 24)
Q279H	1 (0.4)	0 (0.0)	–	–
Q280X	0 (0.0)	1 (0.4)	–	–
Q283X	0 (0.0)	1 (0.4)	–	–
Q312X	0 (0.0)	1 (0.4)	–	–
Q321E	1 (0.4)	0 (0.0)	0 (0.0)	1 (4.2)
Q321R	0 (0.0)	1 (0.4)	–	–
Q321X	0 (0.0)	3 (1.1)	–	–
Q327K	–	–	0 (0.0)	1 (4.2)
Q357X	1 (0.4)	0 (0.0)	–	–
Q386X	3 (1.3)	2 (0.8)	1 (1.8)	0 (0.0)
Q416X	1 (0.4)	0 (0.0)	1 (1.8)	0 (0.0)
Q57X	1 (0.4)	0 (0.0)	–	–
R112C	7 (3.0)	8 (3.0)	–	–
R220X	7 (3.0)	9 (3.4)	2 (3.6)	0 (0.0)
R227Q	4 (1.7)	7 (2.6)	–	–
R227X	6 (2.6)	13 (4.9)	0 (0.0)	2 (8.3)
R301G	6 (2.6)	3 (1.1)	–	–
R301P	1 (0.4)	1 (0.4)	–	–
R301X	6 (2.6)	6 (2.3)	1 (1.8)	1 (4.2)
R342Q	6 (2.6)	2 (0.8)	–	–
R342X	3 (1.3)	4 (1.5)	1 (1.8)	1 (4.2)
R356W	3 (1.3)	3 (1.1)	0 (0.0)	1 (4.2)
R363X	0 (0.0)	1 (0.4)	–	–
R49G	0 (0.0)	1 (0.4)	–	–
R49L	1 (0.4)	0 (0.0)	–	–
R49S	1 (0.4)	0 (0.0)	–	–
S247P	0 (0.0)	1 (0.4)	–	–
S297C	0 (0.0)	1 (0.4)	1 (1.8)	0 (0.0)
S345P	3 (1.3)	2 (0.8)	0 (0.0)	1 (4.2)
S345X	0 (0.0)	1 (0.4)	1 (1.8)	0 (0.0)
S78X	0 (0.0)	1 (0.4)	–	–
splicing	3 (1.3)	2 (0.8)	–	–
V269A	1 (0.4)	1 (0.4)	–	–
V316E	1 (0.4)	1 (0.4)	–	–
W162X	3 (1.3)	0 (0.0)	–	–
W18X	2 (0.9)	0 (0.0)	–	–
W204X	3 (1.3)	5 (1.9)	2 (3.6)	0 (0.0)
W209X	1 (0.4)	0 (0.0)	0 (0.0)	1 (4.2)
W236C	0 (0.0)	3 (1.1)	1 (1.8)	0 (0.0)
W24X	1 (0.4)	3 (1.1)	–	–
W262X	1 (0.4)	0 (0.0)	–	–
W277X	–	–	0 (0.0)	1 (4.2)
W287X	1 (0.4)	1 (0.4)	–	–
W340X	0 (0.0)	2 (0.8)	–	–
W349R	1 (0.4)	2 (0.8)	–	–
W349X	6 (2.6)	1 (0.4)	1 (1.8)	0 (0.0)

Genetic variant	Adult patients		Pediatric patients	
	**Male (*n* = 233)**	**Female (*n* = 266)**	**Male (*n* = 55)**	**Female (*n* = 24)**
W380X	1 (0.4)	0 (0.0)	–	–
W425X	–	–	1 (1.8)	0 (0.0)
W47G	1 (0.4)	0 (0.0)	–	–
W77X	1 (0.4)	0 (0.0)	–	–
W81S	3 (1.3)	3 (1.1)	1 (1.8)	0 (0.0)
Y134S	1 (0.4)	0 (0.0)	0 (0.0)	1 (4.2)
Y151X	1 (0.4)	1 (0.4)	–	–
Y152X	1 (0.4)	0 (0.0)	–	–
Y173X	0 (0.0)	1 (0.4)	–	–
Y216C	1 (0.4)	0 (0.0)	–	–
Y216X	0 (0.0)	1 (0.4)	–	–
Y222X	0 (0.0)	1 (0.4)	–	–
Y365X	1 (0.4)	1 (0.4)	2 (3.6)	0 (0.0)
Y86C	–	–	1 (1.8)	0 (0.0)

Supplementary Table 2 Baseline renal function-related clinical characteristics by A) baseline eGFR (mL/min/1.73 m²) and B) baseline urinary protein (g/24 h).

A)

**Baseline parameter**	**FOS Evaluable Treated Renal cohort (*N* = 1192)**				**Comparator untreated population (*N* = 395) [1]**			
	**Male (*n* = 590)**		**Female (*n* = 602)**		**Male (*n* = 243)**		**Female (*n* = 152)**	
**eGFR (mL/min/1.73 m²)**	**≥ 60 (*n* = 510)**	**< 60 (*n* = 80)**	**≥ 60 (*n* = 531)**	**< 60 (*n* = 71)**	**≥ 60 (*n* = 189)**	**< 60 (*n* = 54)**	**≥ 60 (*n* = 129)**	**< 60 (*n* = 23)**
**Mean age at baseline (SD), years**	38.78 (14.08)	49.00 (11.96)	46.80 (14.22)	60.79 (9.88)	27.3 (11.9)	41.8 (9.9)	38.0 (14.0)	51.9 (12.3)
**Mean urinary protein (SD), g/24 h**	1.15 (11.77)	1.83 (1.84)	0.44 (1.33)	0.63 (0.67)	0.6 (1.0) [*n* = 52]	2.6 (2.3) [*n* = 25]	0.3 (0.4) [*n* = 40]	1.1 (1.5) [*n* = 8]
**Overt proteinuria, *n* (%)**	215 (42.2)	53 (66.3)	191 (36.0)	31 (43.7)	19 (37)	24 (96)	8 (20)	4 (50)
**Mean systolic BP (SD), mmHg**	122.37 (15.63)	125.29 (18.46)	121.74 (17.54)	130.26 (19.56)	125 (15) [*n* = 151]	128 (17) [*n* = 46]	123 (18) [*n* = 98]	135 (18) [*n* = 15]
**Mean diastolic BP (SD), mmHg**	73.50 (10.96)	77.25 (12.03)	72.49 (11.10)	73.67 (11.98)	74 (12)	79 (12)	75 (12)	82 (7)

B)

Baseline parameter	FOS Evaluable Treated Renal cohort (*N* = 1192)						Comparator untreated population (*N* = 90) [1]					
	Male (*n* = 590)			Female (*n* = 602)			Male (*n* = 61)			Female (*n* = 29)		
**Mean urinary protein excretion rate, g/24 h**	**≥ 1.0 (*n* = 76)**	**0.1–< 1.0 (*n* = 211)**	**< 0.1 (*n* = 303)**	**≥ 1.0 (*n* = 41)**	**0.1–< 1.0 (*n* = 196)**	**< 0.1 (*n* = 365)**	**≥ 1.0 (*n* = 22)**	**0.1–< 1.0 (*n* = 21)**	**< 0.1 (*n* = 18)**	**≥ 1.0 (*n* = 5)**	**0.1–< 1.0 (*n* = 17)**	**< 0.1 (*n* = 7)**
**Mean age (SD), years**	42.95 (10.95)	37.41 (13.03)	41.39 (15.46)	51.51 (14.08)	47.35 (13.31)	48.70 (15.11)	38.9 (10.3)	36.0 (12.1)	22.8 (12.8)	47.2 (11.7)	42.3 (12.1)	39.0 (22.0)
**Mean eGFR (SD), mL/min/1.73 m²**	66.85 (29.88)	101.32 (34.06)	104.33 (35.98)	75.74 (28.58)	91.09 (27.52)	95.37 (28.58)	58.5 (25.6)	84.6 (37.1)	138 (56.5)	63.4 (18.9)	89.6 (38.5)	91.9 (41.2)

Baseline is defined as the most recent value within 12 months of the earliest start of agalsidase alfa in FOS, or within 12 months of baseline eGFR assessment in the comparator untreated population.

For patients in the FOS Evaluable Treated Renal cohort, age at baseline was the age at starting agalsidase alfa enzyme replacement therapy.

Overt proteinuria is defined as having > 0.3 g of protein in 24-hour urine collection.

BP, blood pressure; eGFR, estimated glomerular filtration rate; FOS, Fabry Outcome Survey; SD, standard deviation.

Supplementary Table 3 Cardiovascular-related clinical characteristics at baseline.

Baseline parameter, mean (SD)	FOS Evaluable Treated Cardiac cohort (*N* = 724)		Comparator untreated population (*N* = 166) [2]	
	**Male (*n* = 378)**	**Female (*n* = 346)**	**Male (*n* = 66)**	**Female (*n* = 100)**
**Age at baseline, y**	37.46 (16.89)	46.51 (16.70)	33.8 (12.2)	38.0 (18.3)
**BMI, kg/m^2**	22.44 (4.57)	24.43 (5.31)	21.7 (3.8)	23.5 (4.6)
**LVMI, g/m$^{2.7}$**	55.47 (24.55)	54.27 (23.73)	56.8 (27.2)	48.2 (27.2)
**Systolic BP, mmHg**	122.09 (16.20)	121.92 (17.88)	126.5 (16.6)	126.1 (16.0)
**Diastolic BP, mmHg**	72.89 (11.94)	71.68 (11.37)	71.8 (10.3)	71.7 (10.2)
**Heart rate, beats/min**	70.6 (13.1)	69.5 (12.0)	66.7 (11.9)	69.2 (10.7)

Baseline is defined as the most recent value within 12 months of the earliest start of agalsidase alfa in FOS.

BMI, body mass index; BP, blood pressure; FOS, Fabry Outcome Survey; LVMI, left ventricular mass indexed to height; SD, standard deviation.

Supplementary Table 4 Baseline characteristics related to morbidity outcomes.

Parameter	Evaluable Treated Morbidity cohort (*N* = 232)	Comparator untreated population (*N* = 31) [3]
**Male, *n* (%)**	108 (46.6)	27/31 (87)
**Mean age at baseline (SD), years**	54.83 (12.00)	44.3 (9.2)
**Mean weight (SD), kg**	68.75 (15.62)	70.2 (13.3)
**Mean height (SD), cm**	165.53 (10.01)	172.8 (8.2)
**White, %**<br>**Asian, %**	61.6<br>34.1	87<br>3
**Mean urine protein (SD), g/24 h**	0.75 (1.19)	1.1 (1.4)
**Mean urine albumin/ creatinine ratio[a] (SD), mg/g**	260.55 (477.35)	900 (1200)
**Mean plasma creatinine (SD), mg/dL**	1.28 (0.62)	1.6 (0.5)
**Mean eGFR (SD), mL/min/1.73 m^2**	61.16 (15.72)	52.4 (17.7)
**Mean systolic BP (SD), mmHg**	125.24 (19.30)	128 (14)
**Mean diastolic BP (SD), mmHg**	75.06 (12.11)	75 (11)

Baseline is defined as the most recent value within 12 months of the earliest start of agalsidase alfa.

BP, blood pressure; eGFR, estimated glomerular filtration rate; FOS, Fabry Outcome Survey; NR, not reported; SD, standard deviation.

[a] This represents the ratio of albumin (mg/dL) to creatinine (g/dL) in urine.

Supplementary Table 5 Baseline characteristics of A) the populations compared in the mortality analysis and B) FOS patients stratified by survival status.

A)

Characteristic	FOS Evaluable Treated cohort			Comparator untreated population [1]		
	Male patients (*n* = 1025)	Female patients (*n* = 974)	All patients (*N* = 1999)	Male patients (*n* = 279)	Female patients (*n* = 168)	All patients (*N* = 447)
**Mean age at data extraction (range), years**	48.3 (9.6–91.1)	56.4 (12.1–93.3)	52.23 (9.6–93.3)	38.6 (5.0–73.0)	44.9 (10.3–77.1)	41.0 (5.0–77.1)
**Mean age at first symptom (range), years**	18.3 (0.0–72.0)	26.4 (0.0–72.0)	22.00 (0.0–72.0)	10.5 (0.3 –56.0)	17.4 (2.6–56.1)	12.6 (0.3–56.1)
**Mean age at diagnosis (range), years**	29.9 (0.0–78.0)	40.1 (0.0–80.0)	34.82 (0.0–80.0)	23.9 (0–66.5)	29.7 (0–76.2)	26.0 (0–76.2)
**White, n (%)**	670 (66.5)	686 (71.9)	1356 (69.1)	238 (85)	144 (86)	382 (85)

B)

Characteristic	Treated patients who died during agalsidase alfa treatment (*n* = 203)	Treated patients who did not die during agalsidase alfa treatment (*n* = 1968)
**Mean age at onset of symptoms (SD), years**	23.73 (18.69)	22.15 (17.83)
n	146	1248
**Mean age at diagnosis (SD), years**	41.65 (18.73)	34.44 (17.87)
n	196	1875
**Mean age at baseline (SD), years**	52.71 (12.59)	39.89 (16.80)
n	203	1968
**Age group at baseline**		
**Children, *n* (%)**	2 (1.0)	236 (12.0)
**Adults, *n* (%)**	201 (99.0)	1732 (88.0)

**Mean age at last visit (SD), years**	59.99 (12.04)	47.06 (16.64)
n	203	1968
**Disease type**		
**Classic FD, *n* (%)**	37 (75.5)	541 (79.8)
**Non-classic FD, *n* (%)**	12 (24.5)	137 (20.2)
**Mean LVMI at baseline (SD), g/m$^{2.7}$**	78.78 (28.16)	52.98 (23.10)
n	114	972
**LVH status at baseline**		
**LVH, *n* (%)**	100 (87.7)	454 (46.7)
**No LVH, *n* (%)**	14 (12.3)	518 (53.3)
**Mean eGFR at baseline (SD), mL/min/1.73 m^2**	63.70 (33.47)	99.02 (33.72)
n	170	1466
**eGFR category at baseline**		
**< 60 mL/min/1.73 m^2, *n* (%)**	74 (43.5)	169 (11.5)
**≥ 60 mL/min/1.73 m^2, *n* (%)**	96 (56.5)	1297 (88.5)

Baseline is defined as the most recent value within 12 months of the earliest start of agalsidase alfa.

eGFR, estimated glomerular filtration rate; FD, Fabry disease; LVH, left ventricular hypertrophy;

LVMI, left ventricular mass index; SD, standard deviation

Supplementary Table 6 Baseline demographics and characteristics of treated FOS patients with classic and non-classic FD.

Characteristic	Classic, male (*n* = 233)	Classic, female (*n* = 266)	Non-classic (incl. N215S), male (*n* = 104)	Non-classic (incl. N215S), female (*n* = 40)
**Age at onset of symptoms**				
**Mean (SD), years**	14.15 (12.05)	25.61 (16.18)	46.70 (15.41)	36.48 (18.35)
n	185	166	53	21
**Age at diagnosis**				
**Mean (SD), years**	27.35 (13.22)	41.10 (14.85)	51.69 (14.41)	50.00 (14.21)
n	228	257	101	40
**Age at baseline**				
**Mean (SD), years**	35.45 (10.73)	47.31 (13.46)	54.41 (13.24)	53.77 (11.98)
n	233	266	104	40
**Age at last visit**				
**Mean (SD), years**	46.85 (10.86)	55.54 (13.89)	61.45 (12.00)	59.83 (11.68)
n	233	266	104	40
**LVMI at baseline**				
**Mean (SD), g/m$^{2.7}$**	54.76 (19.13)	53.72 (21.06)	68.16 (28.60)	50.28 (17.67)
n	95	143	75	29
**eGFR at baseline**				
**Mean (SD), mL/min/1.73 m^2**	95.13 (33.42)	91.28 (21.23)	80.87 (21.52)	85.74 (25.82)
n	158	205	86	32
**Total FOS-MSSI score**	19.79 (10.44)	17.73 (10.34)	12.15 (6.88)	13.18 (7.25)
n	229	253	99	39

Baseline is defined as the most recent value within 12 months of the earliest start of agalsidase alfa.

eGFR, estimated glomerular filtration rate; FD, Fabry disease; FOS, Fabry Outcome Survey; FOS-MSSI, Fabry Outcomes Survey adjusted Mainz Severity Score Index; LVMI, left ventricular mass indexed to height; SD, standard deviation.

References

[1] R. Schiffmann, et al., Fabry disease: progression of nephropathy, and prevalence of cardiac and cerebrovascular events before enzyme replacement therapy. Nephrol Dial Transplant. 24 (7) (2009) 2102-2111.
[2] C. Kampmann, et al., Onset and progression of the Anderson-Fabry disease related cardiomyopathy. Int J Cardiol. 130 (3) (2008) 367-373.
[3] M. Banikazemi, et al., Agalsidase-beta therapy for advanced Fabry disease: a randomized trial. Ann Intern Med. 146 (2) (2007) 77-86.
